# Cyclin-dependent kinase 2 is an ideal target for ovary tumors with elevated cyclin E1 expression

**DOI:** 10.18632/oncotarget.4600

**Published:** 2015-06-23

**Authors:** Liu Yang, Dongdong Fang, Huijun Chen, Yiyu Lu, Zheng Dong, Han-Fei Ding, Qing Jing, Shi-Bing Su, Shuang Huang

**Affiliations:** ^1^ Research Center for Traditional Chinese Medicine Complexity System, Shanghai University of Traditional Chinese Medicine, Shanghai, China; ^2^ Department of Obstetrics and Gynecology, Zhongnan Hospital of Wuhan University, Wuhan, Hubei Province, China; ^3^ Department of Anatomy and Cell Biology, Medical College of Georgia, Georgia Regents University, Augusta, GA, USA; ^4^ Cancer Center, Georgia Regents University, Augusta, GA, USA; ^5^ Department of Cardiology, Changhai Hospital, Shanghai, China; ^6^ E-institute of Shanghai Municipal Education Committee, Shanghai University of Traditional Chinese Medicine, Shanghai, China; ^7^ Department of Biochemistry and Molecular Biology, Medical College of Georgia, Georgia Regents University, Augusta, GA, USA

**Keywords:** CCNE1, Cdk2, ovarian cancer, tumor development

## Abstract

*CCNE1* gene amplification is present in 15-20% ovary tumor specimens. Here, we showed that Cyclin E1 (CCNE1) was overexpressed in 30% of established ovarian cancer cell lines. We also showed that CCNE1 was stained positive in over 40% of primary ovary tumor specimens regardless of their histological types while CCNE1 staining was either negative or low in normal ovary and benign ovary tumor tissues. However, the status of CCNE1 overexpression was not associated with the tumorigenic potential of ovarian cancer cell lines and also did not correlate with pathological grades of ovary tumor specimens. Subsequent experiments with CCNE1 siRNAs showed that knockdown of CCNE1 reduced cell growth only in cells with inherent CCNE1 overexpression, indicating that these cells may have developed an addiction to CCNE1 for growth/survival. As CCNE1 is a regulatory factor of cyclin-dependent kinase 2 (Cdk2), we investigated the effect of Cdk2 inhibitor on ovary tumorigenecity. Ovarian cancer cells with elevated CCNE1 expression were 40 times more sensitive to Cdk2 inhibitorSNS-032 than those without inherent CCNE1 overexpression. Moreover, SNS-032 greatly prolonged the survival of mice bearing ovary tumors with inherent CCNE1 overexpression. This study suggests that ovary tumors with elevated CCNE1 expression may be staged for Cdk2-targeted therapy.

## INTRODUCTION

Ovarian cancer is the most deadly gynecological malignancy in women, largely due to late diagnosis as tumors have disseminated beyond the ovaries at the diagnosis inover 70% of ovarian cancer patients. In these cases, debulking with chemotherapy is the standard treatment procedure and yields a response rate of more than 80%. However, almost all patients relapse and this clearly highlights the necessity to develop drugs useful for recurrent diseases [1].

Recent studies including The Cancer Genome Atlas (TCGA) reveal that ovarian cancers, especially high grade serous ovarian cancers (HGSOC), are marked by profound chromosomal aberrations (gene amplification and loss) rather than recurrent somatic mutations [2–4]. Besides near-ubiquitous TP53 mutation, point mutations are relatively uncommon at least in HGSOC [4]. Instead, HGSOCs contain widespread DNA copy number changes and one of the most frequent gene amplifications is *CCNE1* which occurs in at least 20% of HGSOC [2, 5, 6]. Importantly, *CCNE1* gene amplification correlates with CCNE1 overexpression in ovarian cancer and appear to have poorer disease-free and overall survival [6]. Immunohistochemistry studies with both primary and metastatic ovary tumor specimens further show that the abundance of cyclin E1 (CCNE1) correlates with tumor progression and predicts a poor prognosis in ovarian cancer patients [7–10]. Taken together, these findings highlight the importance of CCNE1 in ovary tumorigenesis.

CCNE1 mainly coordinates with Cdk2 to facilitate G1/S progression of cell cycle [11]. In ovarian cancer cells, enforcing CCNE1 expression stimulates cell proliferation [6] and increases colony formation [12]. *CCNE1* gene amplification-associated CCNE1 overexpression has been linked to the development of chemo-resistance in ovarian cancer [13, 14]. A recent study further shows that CCNE1 deregulation occurs early in fallopian tube secretory epithelial cell (FTSEC) transformation which promotes the formation of HGSOC [15]. Although all these findings implicate CCNE1 as a promising therapeutic target for at least the set of ovary tumors with elevated CCNE1 expression, developing small molecules to target CCNE1 directly is unlikely because CCNE1 acts as a regulatory subunit of cyclin-dependent kinase (Cdk) complex rather than as an enzyme or receptor. As ovary tumors with elevated CCNE1 level often exhibit higher Cdk2 expression [5, 15] and most of CCNE1-associated tumor promoting effects require the participation of Cdk2 [16], we reasoned that targeting Cdk2 may be an attractive alternative given the current availability of small molecule Cdk2 inhibitors.

The objective of this study was to investigate the potential of Cdk2 inhibitor to suppress ovary tumor progression. With a panel of established ovarian cancer cell lines, we found that majority of ovarian cancer cells lines with CCNE1 overexpression possessed *CCNE1* gene amplification. Immunohistochemistry study with primary ovary tumor specimens showed that over 40% of ovary tumor specimens were positive for CCNE1 staining; in contrast, CCNE1 staining was either negative or very low in normal ovary and benign ovary tumor specimens. However, the status of elevated CCNE1 expression was not relevant to the properties of cell growth and metastatic colonization in ovarian cancer cell lines while CCNE1 staining was not associated with pathological grades of all three histological types of ovarian cancer (serous, mucinous and endometrioid). Despite lack of clear association between CCNE1 expression and tumorigenic behaviors, CCNE1 is critical for the growth of ovarian cancer cell lines with elevated CCNE1 expression because knockdown of CCNE1 diminished the growth of cells with CCNE1 overexpression but not cells without CCNE1 overexpression. To determine the effect of Cdk2 inhibitor on ovarian cancer cell growth, we showed that ovarian cancer cells with elevated CCNE1 expression are at least 40 times more sensitive to Cdk2 inhibitor SNS-032 than those without CCNE1 overexpression, immortalized OECs and FTSECs. Finally, we demonstrated that SNS-032 effectively suppressed the tumorigenecity of ovarian cancer cells with elevated CCNE1 expression by prolonging the survival of animals bearing tumors derived from ovarian cancer cells with elevated CCNE1 expression and inhibiting peritoneal metastatic colonization.

## RESULTS

### CCNE1 expression in established ovarian cancer cell lines

**Figure 1:**
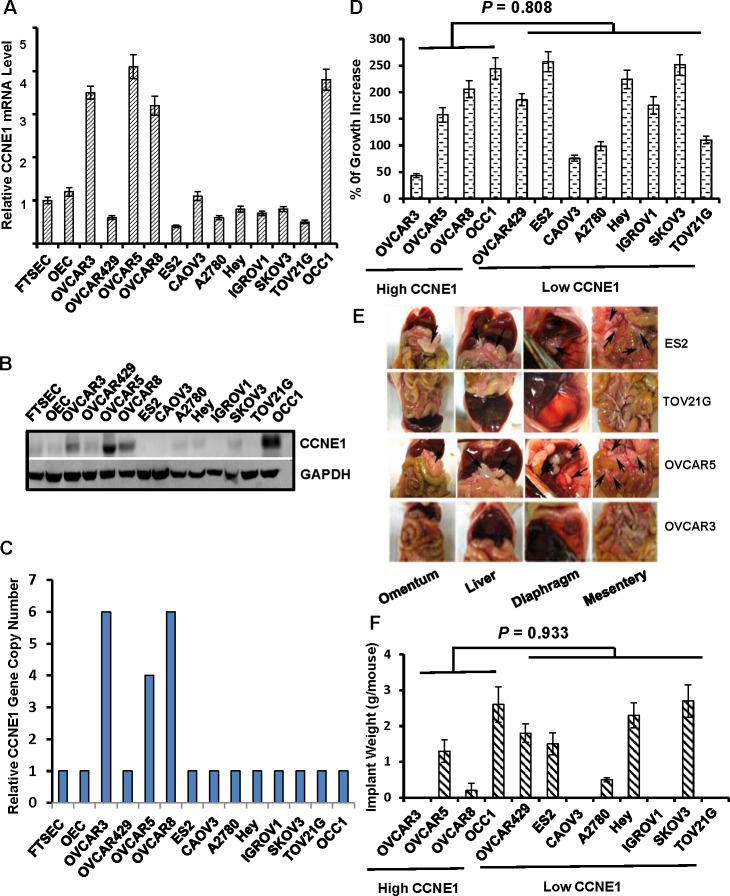
The status of CCNE1 overexpression is not associated with tumorigenic behaviors in established ovarian cancer cells **A.**Total RNA was isolated from overnight-cultured cells and subjected to qRT-PCR to quantitate the amount of CCNE1 mRNA. β-actin mRNA was used as an internal standard for normalization. **B.** Overnight-cultured cells were harvested and cell lysates were subjected to immunoblotting to detect CCNE1 protein using CCNE1 mAb. Membrane was stripped and reprobled with GAPDH polyclonal antibody for loading normalization. **C.** Genomic DNA was isolated from overnight-cultured cells and subjected to qPCR to analyze copy number of *CCNE1* gene. *ACTB* gene was used for normalization. **D.** Cells (50,000/well) were plated in 24-well plates for overnight and then cultured for 2 days followed by MTT assay. % of growth increase was calculated as [(OD_overnight_–; OD_end_)/OD_overnight_] x 100. **E.** Cells (1×10^7^cell/mouse) from various ovarian cancer lines were intraperitoneally injected to nude mice for 4 weeks to allow metastatic colonization. Images are the views of various areas in peritoneal cavity. Arrows point to metastatic implants. **F.** Metastatic implants were collected and weighed. Data are means ± SE. *n* = 6.

Elevation of CCNE1 level has been reported in various histological types of human ovarian tumors including HGSOC [5, 7]. Integrated analysis of ovarian carcinoma from the study of TCGA further showed that*CCNE1* gene is amplified in 15-20% of HGSOC [4]. To determine if elevated CCNE1 expression is linked to *CCNE1* gene amplification in ovarian cancer, we initially examined the level of CCNE1 mRNA and protein in a panel of established ovarian cancer cell lines, immortalized ovary epithelial cells (OECs) and FTSECs. The abundance of CCNE1 mRNA and protein were generally correlated in all cell lines examined (Figure 1A and 1B). Level of CCNE1 was elevated in OVCAR3, OVCAR5, OVCAR8 and OCC1 cells compared to that in OECs or FTSECs whereas the remaining cell lines displayed either similar or lower level of CCNE1 compared to OECs and FTSECs (Figure 1A and 1B). We subsequently isolated genomic DNA and performed qPCR to analyze the copy number of *CCNE1* gene in these cell lines. Comparing to that in OECs or FTSECs, *CCNE1* gene was not amplified in ovarian cancer cell lines without CCNE1 overexpression (Figure 1C). Among lines with elevated CCNE1 expression, *CCNE1* gene amplification was detected in OVCAR3, OVCAR5 and OVCAR8 cell lines (Figure 1C). However, relative copy number of *CCNE1* gene in OCC1 cells was the same as that in OECs and FTSECs (Figure 1C). Our data show that *CCNE1* gene amplification is present in majority of ovarian cancer cell lines with elevated CCNE1 overexpression, thus indicating that *CCNE1* gene amplification is at least one of the principal factors contributing to CCNE1 overexpression in ovarian cancer.

To investigate whether the status of elevated CCNE1 expression could be linked to tumorigenic behaviors of ovarian cancer cell lines, we first assessed cell growth of these cell lines. MTT assay showed an increase of 43% to 257% in 2-day growth period among these lines (Figure 1D). However, we were unable to detect an apparent association between the abundance of CCNE1 and growth rate among them (*P* = 0.808 between with and without CCNE1 overexpression). Subsequent peritoneal metastatic colonization revealed that some high CCNE1 expressers were capable of undergoing efficient metastatic colonization while others were not (Figure 1E and 1F). Similarly, ovarian cancer cell lines without CCNE1 overexpression could also be either metastatic or non-metastatic (Figure 1E and 1F; *P* = 0.933between with and without CCNE1 overexpression). These data thus failed to establish a correlation between the status of CCNE1 overexpression and tumorigenic potential in ovarian cancer cell lines.

### CCNE1 expression in human ovarian tumors

**Figure 2:**
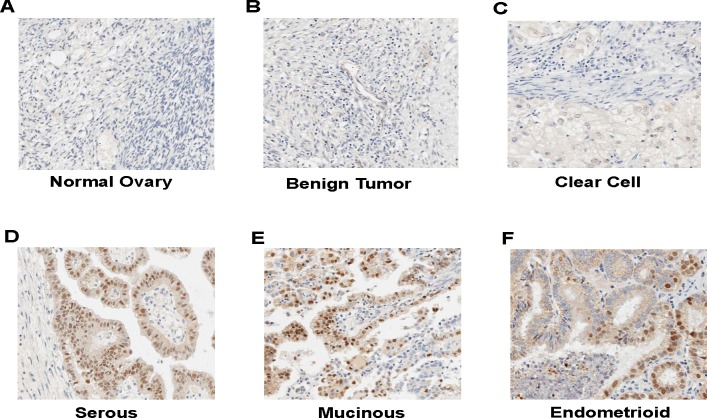
Immunohistochemistry of CCNE1 on ovary tumor specimens **A.** Normal ovary tissue. **B.** Benign ovary tumor tissue. **C.** Clear cell type of ovary tumor specimen. **D.** Serous type of ovary tumor specimen. **E.** Mucinous type of ovary tumor specimen. **F.** Endometrioid type of ovary tumor specimen.

Previous studies reveal that *CCNE1* gene amplification correlates with tumor progression and predicts a poor prognosis in ovarian cancer patients [7, 9, 17]. However, our *in vitro* studies with established ovarian cancer cell lines failed to establish a correlation between CCNE1 expression and tumorigenic potential (Figure 1D, 1E and 1F). To understand this differentiation, we examined CCNE1 staining in human ovarian tumor specimens by performing immunohistochemistry on a tissue array that contained normal, benign and ovarian tumor tissues. CCNE1 staining was negative or low in all normal ovary and benign tumor tissues (Table 1, Figures 2A, S1A, 2B and S1B). The only clear cell type ovary tumor specimen showed low CCNE1 staining (Table 1 and Figure 2C). In contrast, 43.8% of serous (21/48), 55.6% of mucinous (10/18) and 40.0% of endometrioid ovary tumor specimens (12/30) were strong for CCNE1 staining (Table 1, Figures 2D, S1C, 2E, S1D, 2F and S1E). The staining of CCNE1 was not associated with histological types or correlated with pathological grades of the disease (Table 1). These results were consistent with the data generated from ovarian cancer cell lines, in which CCNE1 level is found not to be correlated with the tumorigenic potential of ovarian cancer cell lines (Figure 1).

**Table 1:** Correlation between the status of CCNE1 staining and clinicopathological parameters of ovarian cancer patients

	Total No. of patients	CCNE1 Staining		*P* value
		Negative or Low (%)	Positive (%)	
Histological types				0.565
Serous	48	27 (56.3%)	21 (43.7%)	
Mucinous	18	8 (44.4%)	10 (55.6%)	
Endometrioid	30	18 (40.0%)	12 (60.0%)	
Pathologic Grade				0.582
I	30	15 (50.0%)	15 (50.0%)	
II	27	14 (51.9%)	13 (48.1%)	
III	39	24 (47.4%)	15 (52.6%)	

### Presence of CCNE1 is critical for growth of ovarian cancer cells with elevated CCNE1 expression

**Figure 3:**
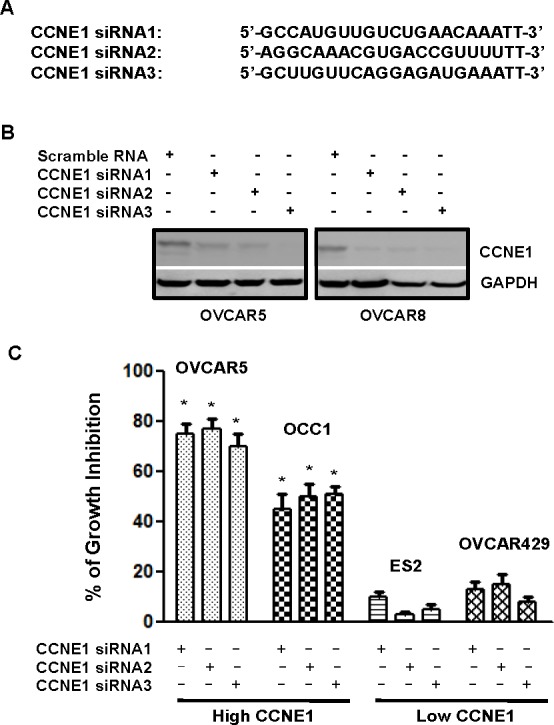
Ovarian cancer cells with elevated CCNE1 expression is sensitive to CCNE1 knockdown **A.** Sequences of CCNE1 siRNAs. **B.** OVCAR5 and OVCAR8 cells were transfected with scrambled control or CCNE1 siRNAs for 3 days, then lysed and lysates were subjected to immunoblotting to detect CCNE1 with CCNE1 mAb. Membrane was stripped and reprobed with GAPDH polyclonal antibody for loading normalization. **C.** Cells (50,000 cells/well of 24-well plate) were transfected with scrambled control or CCNE1 siRNAs for overnight, then re-fed with fresh medium and cultured for 4 days prior to MTT assay to assess cell growth. % of growth inhibition = [(OD_control_-OD_siRNA_)/OD_control_] x 100. Data are means ± SE. *n* = 4. *, *P* < 0.005 *vs* scrambled control.

Although we were unable to detect clear correlation between CCNE1 and tumorigenic potential of ovarian cancer cells (Figure 1), the observation of elevated CCNE1 expression in over 40% ovary tumor specimens (Table 1 and Figure 2) raised the possibility that the presence of CCNE1 may be only involved in the tumorigenecity of ovarian cancer cells with elevated CCNE1 expression. To test this possibility, we designed three siRNAs that target various regions of CCNE1 mRNA (Figure 3A). Immunoblotting to detect CCNE1 showed that all three CCNE1 siRNAs effectively diminished the level of CCNE1 in OVCAR5 and OVCAR8 cells when compared with the scrambled control (Figure 3B). With these siRNAs, MTT assay showed that knockdown of CCNE1 led to 65-90% of reduction in cell growth in cell lines with elevated CCNE1 expression (OVCAR3, OVCAR5, OVCAR8 and OCC1) (Figure 3C and S2). However, CCNE1 siRNAs did not significantly alter the growth of ovarian cancer cell lines without CCNE1 overexpression (ES2, OVCAR429, IGROV1, SK-OV3) (Figures 3C and S2). Since *CCNE1* gene is not amplified in OCC1 cells (Figure 1C), these results suggest that the presence of CCNE1 is critically important for the growth of ovarian cancer cells with elevated CCNE1 expression regardless of *CCNE1* gene amplification status.

### Ovarian cancer cells with elevated CCNE1 expression are greatly more sensitive to Cdk2 inhibitor

**Figure 4:**
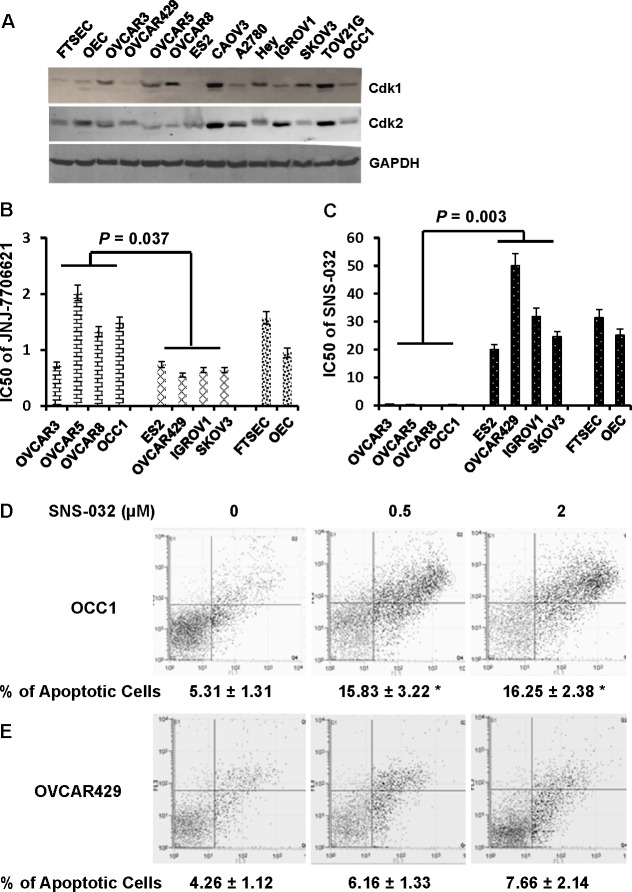
Cdk2 inhibitor SNS-032, but not Cdk1 inhibitor JNJ-7706621 selectively inhibits growth of ovarian cancer cells with elevated CCNE1 expression by inducing apoptosis **A.** Overnight-cultured cells were harvested and cell lysates were subjected to immunoblotting to detect Cdk1 and Cdk2 protein using the respective antibodies. Membrane was stripped and reprobled with GAPDH polyclonal antibody for loading normalization. **B, C.** Ovarian cancer cells (40,000 cells/well) were plated in 24-well plates for overnight and varying concentrations of JNJ-7706621 **B.** or SNS-032 **C.** were added to cells for 4 days. Cell growth was evaluated by MTT assay and IC50 was estimated as the concentration required for reach 50% growth reduction comparing to untreated cells. **D, E**. OCC1 **D.** or OVCAR429 cells **E.** were treated with 0.5 or 2μM SNS-032 for 2 days, then stained with Annexin V-FITC/propidium iodide and analyzed by FACS. Data are means ± SE. *n* = 4. *, *P* < 0.005 *vs* 0 μM of SNS-032.

CCNE1 forms a complex with and functions as a regulatory subunit of Cdk2 to regulate cell cycle G1/S transition [11]. We initially performed immunoblotting to detect Cdk1 and Cdk2 in ovarian cancer cell lines. Levels of Cdk1 and Cdk2 varied in these cell lines (Figure 4A). However, we failed to detect a correlation between the abundance of CCNE1 and Cdk1 or Cdk2. Recent study showed that ovarian cancer cells overexpressing CCNE1 exhibited greater Cdk2 activity [12]. We thus hypothesized that ovarian cancer cells with elevated CCNE1 expression would be more sensitive to the suppression of Cdk2 activity. To test this hypothesis, cell lines with and without elevated CCNE1 expression were treated with varying concentration of selective inhibitor to Cdk1 JNJ-7706621 [18] or Cdk2 SNS-032 [19]. MTT assay showed that OVCAR429 was the most sensitive line to JNJ-7706621 among ovarian cancer cell lines without CCNE1 overexpression which had an IC50 of 0.55 μM while OVCAR5 was the least sensitive line among ovarian cancer cell lines with elevated CCNE1 expression which had an IC50 of 2.02 μM (Figure 4B). This reveals a less than 4-fold difference in the sensitivity to JNJ-7706621 between cell lines with and without CCNE1 overexpression (*P* = 0.037 between with and without CCNE1 overexpression). In contrast, IC50 of SNS-032 in ES2, the most sensitive ovarian cancer cell line among those without CCNE1 overexpression was 20.05 μM while IC50 of SNS-032 in OVCAR3, the least sensitive one among those with elevated CCNE1 expression was 0.53 μM (Figure 4C). This uncovers a 40-fold difference in the sensitivity to Cdk2 inhibitor between ovarian cancer cell lines with and without CCNE1 overexpression (*P* = 0.003). In a parallel experiment, we further analyzed the sensitivity of immortalized OECs and FTSECs to SNS-032. IC50s of OECs and FTSECs in a 4-day treatment period were 25.21 and 31.44 μM respectively (Figure 4C), resembling to those observed in ovarian cancer cells without CCNE1 overexpression.

To determine whether SNS-032-led cell growth suppression was the consequence of cell apoptosis, OCC1 and OVCAR429 cells were treated with 0.5 or 2 μM of SNS-032 for 2 days followed by Annexin V staining-based flow cytometry to detect apoptotic cells (Figure 4D and 4E). Treatment of SNS-032 resulted in a significant increase in the percentage of apoptotic cells in OCC1, a line with elevated CCNE1 expression (Figure 4D). In contrast, identical concentration of SNS-032 only marginally increased the percentage of apoptotic cells in OVCAR429, a line without CCNE1 overexpression (Figure 4E). These results demonstrate that Cdk2 inhibitor SNS-032 selectively induces apoptosis in ovarian cancer cells with elevated CCNE1 expression.

### Cdk2 inhibitor prolongs the survival of mice bearing tumors derived from cells with elevated CCNE1 expression

**Figure 5:**
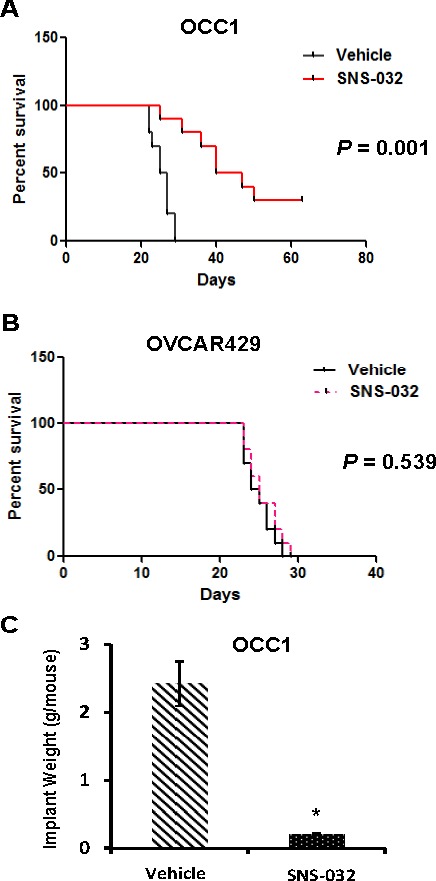
SNS-032 suppresses tumorigenecity of ovarian cancer cells with CCNE1 overexpression **A., B.** Kaplan-Meier curve summarizing survival of mice injected with OCC1 **A.** and OVCAR429 cells **B.**. Female athymic nude mice were injected *i.p.* with OCC1 or OVCAR429 cells (1 × 10^7^ cells/mouse) for 5 days followed by administration of 30 mg/kg SNS-032twice per week until animal died. **C**. OCC1 cells (1 × 10^7^ cells/mouse) were injected *i.p.* to nude mice for 5 days followed by SNS-032 treatment for 3 weeks. Mice were sacrificed and metastatic implants were collected/weighed. Data are means ± SE. *n* = 10. *, *P* < 0.001 *vs* vehicle.

The observation that Cdk2 inhibitor SNS-032 selectively induces apoptosis in ovarian cancer cells with elevated CCNE1 expression prompted us to investigate its efficacy to suppress ovarian tumor progression with the well-established ovarian tumor peritoneal metastatic colonization model [20, 21]. Female athymic nude mice were intraperitoneally injected with OCC1, OVCAR429, ES2 or OVCAR5 cells and then received SNS-032 twice a week starting 5 days after tumor cell injection. Mice receiving any of these cell lines died between 4 to 6 weeks (Figures 5A, 5B, S3A and S3B). Administering SNS-032 slightly increased the lifespan of mice receiving OVCAR429 cells while exhibited no improvement on mice injected with ES2 cells (Figure 5B and S3B). In contrast, SNS-032 greatly prolonged the survival of mice injected with OCC1 and OVCAR5 cells (Figure 5A and S3A). In fact, 30% of OCC1 cell-injected mice treated with SNS-032 survived longer than 9 weeks while all OCC1 cell-injected mice receiving vehicle died within 4 weeks (Figure 5A). These results show that SNS-032 promotes the survival of mice bearing tumors derived from ovarian cancer cells with elevated CCNE1 expression but not mice bearing tumors derived from cells without CCNE1 overexpression.

In a parallel set of experiment, we determined the effect of SNS-032 on metastatic colonization. Mice were injected with OCC1 cells for 5 days, and then administrated with vehicle or SNS-032 for 3 weeks followed by collection metastatic implants from sacrificed animals. Measurement of implant weight showed that mice receiving SNS-032 displayed an average of 85% less metastatic implants than control mice (administered with vehicle) (Figure 5C). These results suggest that prolonged survival of tumor-bearing mice is most likely the consequence of suppressed metastatic colonization.

## DISCUSSION

CCNE1 is abnormally expressed in various tumor types [11, 22]. Its role in tumorigenesis has been well established in various tumor models [15, 23]. In ovarian cancer, *CCNE1* gene was amplified in approximately 20% of ovary tumor specimens including both serous and endometrioid types [2, 4, 5]. Immunohistochemistry studies further indicate that CCNE1 overexpression may contribute the malignancies of ovary tumors [7, 9]. Here, we show that elevated CCNE1 expression is detected in more than 30% of the established ovarian cancer cell lines (Figure 1A and 1B) and majority of the lines with CCNE1 overexpression displayed *CCNE1* gene amplification (Figure 1C). Our results support the notion that *CCNE1* gene amplification is at least one of the key factors contributing to elevated CCNE1 expression in ovarian cancer.

Consistent with published immunohistochemistry studies [7, 9, 24], we showed that over 40% of primary ovary tumor specimens were positive for CCNE1 staining (Table 1 and Figure 2). Although CCNE1 staining was negative or low in all normal ovary tissues or benign ovary tumor specimens, we did not detect a correlation between CCNE1 staining and pathological grades in ovary tumor specimens (Table 1). This result concurs with the observation that the status of CCNE1 overexpression was not associated with growth rate and metastatic potential among the established ovarian cancer cell lines (Figure 1). An early study reported that high CCNE1 expression is a significant and independent predictor for prolonged overall survival in late stage ovarian cancer patients [10]. Although data from our studies do not support CCNE1 as a key factor to promote tumorigenic behaviors including cell growth and metastatic potential in ovarian cancer cells, it does not rule out the potential of CCNE1 as a potent tumor-promoting factor in ovarian cancer cells with elevated CCNE1 expression. In fact, forced CCNE1 expression accelerates cell growth and increases chemosensitivity in ovarian cancer cells [6, 13]. We also showed that knockdown of CCNE1 led to the suppression of cell growth of ovarian cancer cells with inherently elevated CCNE1 expression (Figure 3).

Although we failed to detect a clear correlation between CCNE1 level and malignancies in both established ovarian cancer cell lines as well as primary tumor specimens (Figures 1 and 2), the frequent CCNE1 gene amplification in ovarian cancer and the ability of CCNE1 to facilitate the formation of HGSOC from FTSECs indicates that ovarian cancer cells with elevated CCNE1 expression could have developed addiction to CCNE1 overexpression for cell growth/survival. This possibility is clearly supported by our observation that the growth of ovarian cancer cell lines with CCNE1 overexpression are greatly inhibited by CCNE1 knockdown while CCNE1 siRNAs did not significantly alter the growth of lines without CCNE1 overexpression (Figure 3). Our results are also in agreement with two recent studies reporting that depleting CCNE1 leads to the suppression of ovarian cancer cell proliferation [6, 16]. Oncogene addiction is dependence on oncogene even though this oncogene is not needed before its activation [25, 26]. Some of the well-established examples on oncogene addiction include amplification of HER2 in breast cancer [27], mutated EGFR in non-small cell lung cancer [28], mutated BRAF in melanomas [29] and Bcr-Abl in chronic myeloid leukemia [30, 31]. Our finding that some ovarian cancer cells are addicted to the presence of CCNE1 indicates that CCNE1 may be a driver oncogene to initiate ovarian cancer.

Interfering with CCNE1 function may be an effective strategy to suppress CCNE1-overexpressing ovary tumors, the nature of CCNE1 as a regulatory subunit of CDK complex rather than as an enzyme or receptor indicates that CCNE1 itself is not likely to be an ideal drug target. Because CCNE1 facilitates cell cycle transition mainly by forming complex with Cdk2 (CCNE1 may also complex with Cdk1 and Cdk4 to a lesser extent) [11], we speculated that blocking CCNE1 function may be achieved by targeting the Cdks that interact with CCNE1. In this study, we show that ovarian cancer cell lines with elevated CCNE1 expression are at least 40 times more sensitive to Cdk2 inhibitor SNS-032 than lines without inherent CCNE1 overexpression, non-cancerous OECs and FTSECs (Figure 4). SNS-032 apparently inhibits cell growth of inherently CCNE1-overexpressing ovarian cancer cells by inducing apoptosis (Figure 4). Since Cdk1 inhibitor JNJ-7706621 was not as selective as SNS-032 in suppressing cell growth between ovarian cancer cell lines with and without elevated CCNE1 expression (Figure 4), our results raise the possibility that a subset of ovarian cancer patients with elevated CCNE1 level may be helped by Cdk2 inhibitors. This possibility is supported by our observation that Cdk2 inhibitor SNS-032 suppressed metastatic colonization of CCNE1-overexpressing ovarian cancer cells and greatly prolonged the survival of mice bearing ovary tumors with CCNE1 overexpression (Figure 5). In conclusion, our study has laid a foundation on using currently available Cdk2 inhibitor for ovary tumors that exhibit elevated CCNE1 expression.

## MATERIALS AND METHODS

### Cells, siRNAs and other reagents

Immortalized human ovarian epithelial cells and immortalized human fallopian tube secretory epithelial cells were purchased from ABM (Richmond, BC, Canada) and maintained according to manufacturer's protocol. All human ovarian cell lines were maintained in DMEM containing 10% FCS at 37°C in a humidified incubator supplied with 5% CO_2_. All siRNAs were purchased from Shanghai GenePharma (Shanghai, China). Cdk1 inhibitor JNJ-7706621 and Cdk2 inhibitor SNS-032 (BMS-387032) were purchased from Selleckchem (Houston, TX). Information for primer sequences is included in Supplementary Data.

### qRT-PCR and qPCR

Total RNA was extracted from cells using Trizol (Life Technologies, Carlsbad, CA), treated by DNaseI and reverse transcribed with SuperScriptase II (Life Technologies). Generated cDNA was subjected to quantitative PCR to measure CCNE1 and β-actin mRNA levels with specific primer sets. The expression levels were standardized by comparing the Ct values of target to that of β-actin mRNA. To measure copies of *CCNE1* gene in cells, genomic DNA was isolated using DNAzol (Life Technologies) and used as template for qPCR. The copy number of *CCNE1*gene was standardized to β-actin gene.

### Immunoblotting

To determine the amount of CCNE1, Cdk1 or Cdk2 in cells, overnight-cultured cells were harvested using radio-immunoprecipitation assay (RIPA) buffer. To determine the effect of CCNE1 siRNAs on CCNE1 expression, cells were transfected with scrambled control or CCNE1 siRNAs for 3 days and then harvested using RIPA. Cellular proteins were resolved by 12% SDS-PAGE, transferred to nitrocellulose membrane and blocked before probing with anti-CCNE1 mAb (Cell Signaling Technology, Danvers, MA), anti-Cdk2 mAb (Life Technologies), Cdk2 mAb (Cell Signaling Technology) or anti-GAPDH polyclonal antibody (Santa Cruz Biotechnology, Santa Cruz, CA).

### Histochemistry

CCNE1 level in ovary tumors and normal ovary tissues were evaluated by IHC using anti-CCNE1 mAb on commercial tissue arrays (Super Biotek, Shanghai, China) as previously described [32, 33]. The array contained 10 normal ovary tissues, 10 benign ovarian tumor specimens and 97 ovarian tumor samples (1 clear cell, 48 serous, 18 mucinous and 30 endometrioid). Extent of immunostaining was given a modified histochemical score (MH-score) that considers both the intensity and the percentage of cells stained at each intensity. Samples with below the average MH-score were considered as no/weak staining while samples with above the average MH-score considered as strong staining.

### MTT assay

Cell growth was assayed by MTT as described previously [34]. Briefly, 5×10^4^ cells were seeded into 24-well culture plates and allowed to grow for 2-4 days prior to the addition of MTT. Dilutions of pharmacologic agents in growth media were done in four replicate rows per cell type and per dilution. Plates were then incubated in a humidified incubator in 5% CO2 at 37°C. At the time points indicated, 100 μL of MTT solution (5 mg/mL MTT in PBS) were added to a total volume of 1 ml and incubated in 5% CO_2_ at 37°C for 4 h. Formazan crystals were dissolved with 0.5 ml DMSO and absorbance at 570 nm was determined with a plate reader. To determine the effect of CCNE1-knockdown on cell growth, cells were transfected with CCNE1 or control siRNAs for 4 days prior to MTT assay. Growth inhibition was calculated with the formula of [(absorbance of treated –; absorbance of control)/(absorbance of control)] x 100%.

### Apoptosis detection

Apoptosis was quantified using an Annexin V-FITC detection kit (BD Biosciences, San Jose, CA) according to manufacturers’ instructions. Briefly, cells were treated with 0.5 or 2 μM Cdk2 inhibitor for 2 days, then trypsinized and resuspended in binding buffer (100 mM HEPES, 1.4 M NaCl, 25 mM CaCl_2_, pH 7.4) containing Annexin V-FITC and propidium iodide. Stained cells were analyzed by fluorescence activating cell sorter (FACS) (Becton Dickinson, CA, USA) and the percentage of apoptotic cell population was determined using ModFit LT 3.0 software (Becton Dickinson, CA, USA).

### Peritoneal metastatic olonization assay

Peritoneal metastatic colonization assays were performed as previously described [20]. Female athymic nude mice (BALB/c, 6 weeks of age) were obtained from Shanghai Laboratory Animal Research Center (Shanghai, China). To determine metastatic potential of each ovarian cancer cell line, cells grown in log-phase were resuspended in PBS and intraperitoneally injected into nude mice at 10^7^cells/mouse. Three weeks after injection, the mice were sacrificed and autopsied. Visible metastatic implants were collected and weighed. To determine the effect of SNS-032 in peritoneal metastatic colonization, nude mice were injected with ES2, OCC1, OVCAR429 and OVCAR5 cells (10^7^cells/mouse), and SNS-032 dissolved in 5% Propylene glycol was administered by intraperitoneal injection twice per week at 30 mg/kg 5 days post-operation. A subset of mice receiving OCC1 cells were euthanized 3 weeks post SNS-032 treatment and autopsied. Visible metastatic implants were collected and weighed. Animal housing and experimental conditions were in compliance with the protocol approved by the Institutional Animal Care and Use Committee at the Shanghai University of Traditional Chinese Medicine.

### Statistical analysis

Statistical analyses of cell growth, metastatic implant weights and CCNE1 mRNA levels were performed by ANOVA and student *t* test. Chi-square test was used to compare covariates between CCNE1 staining and clinicopathological parameters. Mann-Whitney test was used to analyze the significance in mouse survival. Statistical analyses were aided by SPSS (release 15.0; SPSS Inc). *P* < 0.05 was considered to be significant.

## SUPPLEMENTARY MATERIAL FIGURES


