# Supplementary material for: Cyclin-dependent kinase 2 is an ideal target for ovary tumors with elevated cyclin E1 expression
Source: Oncotarget. 2015 Jun 23;6(25):20801–12. doi: 10.18632/oncotarget.4600 (PMC4673230; doi:10.18632/oncotarget.4600)
Supplement: Supplementary file 1 [file oncotarget-06-20801-s001.pdf]

Cyclin-dependent kinase 2 is an ideal target for ovary tumors with elevated cyclin E1 expression

Supplementary Material

SUPPLEMENTAL INFORMATION

Primers for CCNE1

Forward: AGCGGTAAGAAGCAGAGCAG

Reverse: CGCTGCAACAGACAGAAGAG

Primers for β-actin

Forward: 5'-GTGCTCAGGGCTTCTTGTCCTTT-3'

Reverse: 5'-TTTCTCCATGTCGTCCCAGTTGGT-3'

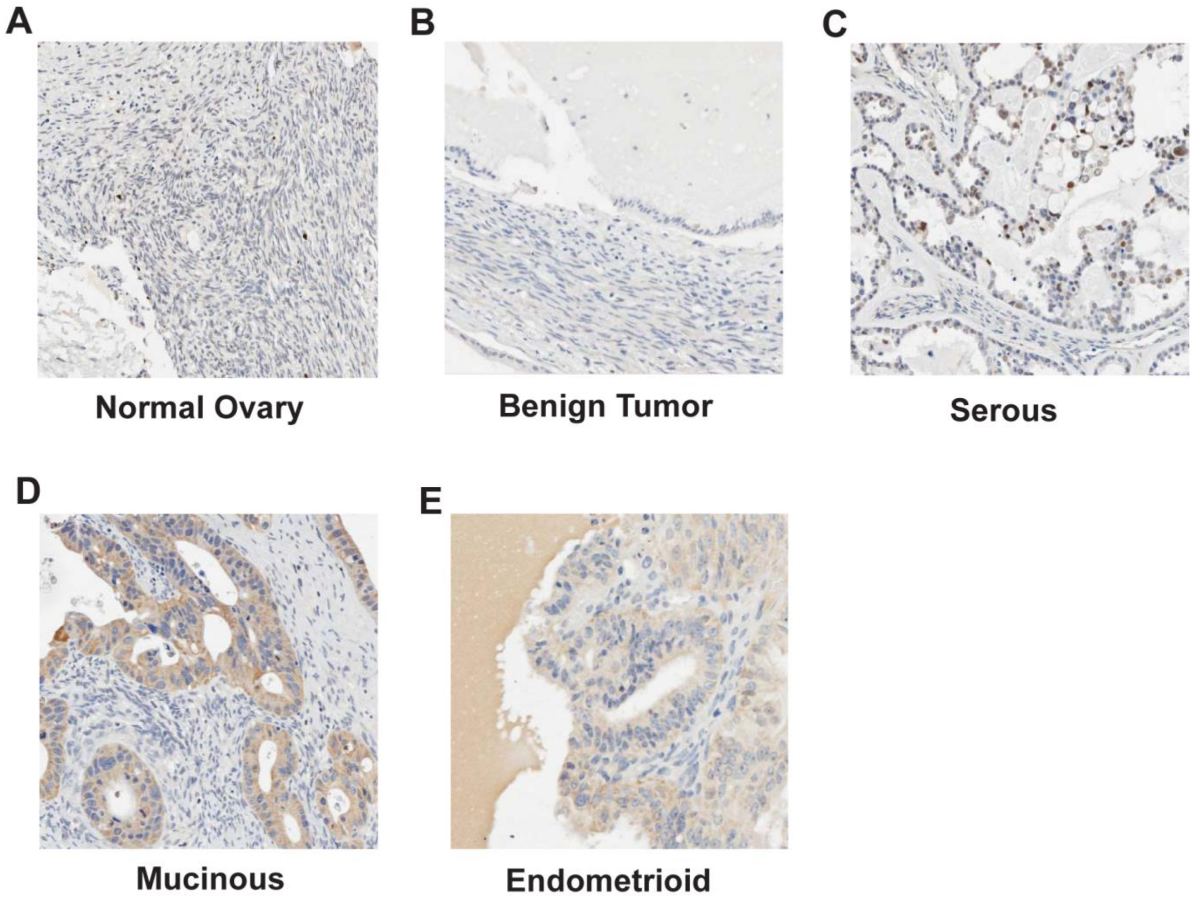

Figure S1. Immunohistochemistry of CCNE1 on ovary tumor specimens. ***A.*** Normal ovary tissue. ***B.*** Benign ovary tumor tissue. ***C.*** Serous type of ovary tumor specimen. ***D.*** Mucinous type of ovary tumor specimen. ***E.*** Endometrioid type of ovary tumor specimen.

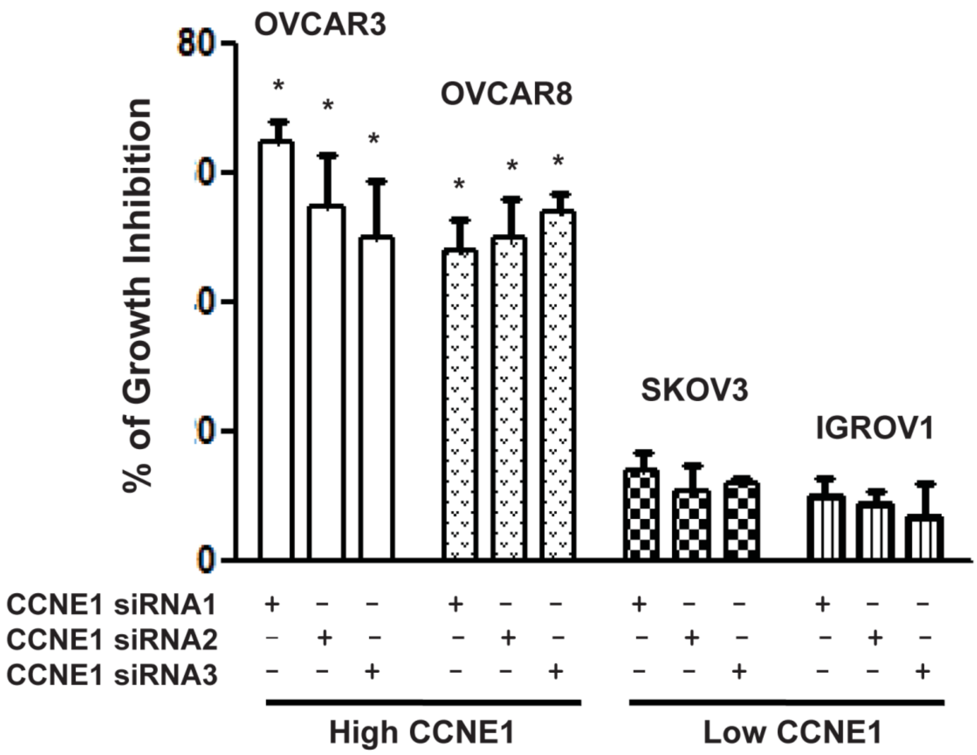

Figure S2. Ovarian cancer cells with elevated CCNE1 expression is sensitive to CCNE1 knockdown. Cells were transfected with scrambled control or CCNE1 siRNAs for overnight, then re-fed with fresh medium and cultured for 4 days prior to MTT assay to assess cell growth. % of growth inhibition = $[(OD_{control}-OD_{siRNA})/OD_{control}]$ x 100. Data are means ± SE. n=4. *, $P < 0.005$ *vs* scrambled control.

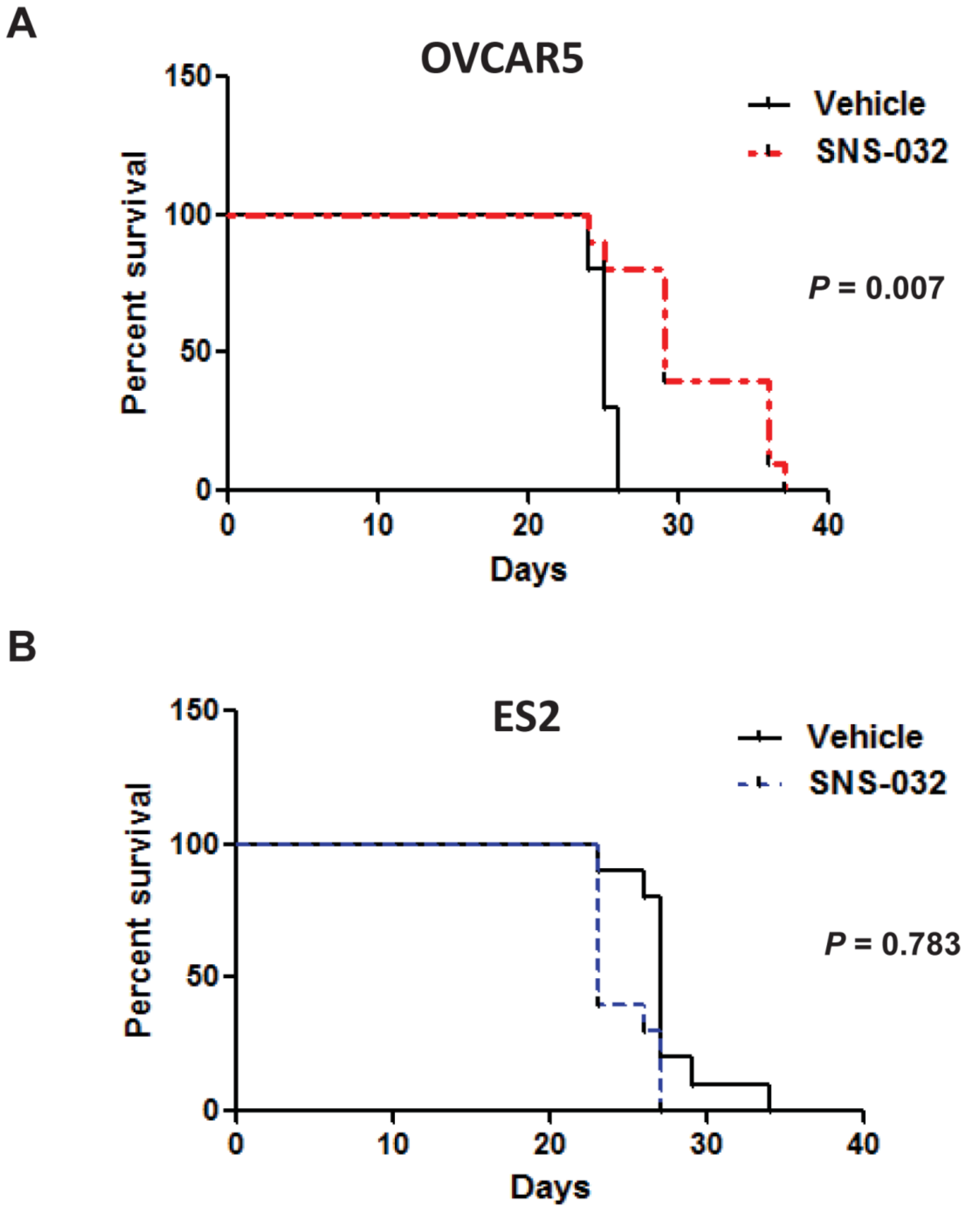

Figure S3. SNS-032 prolongs the survival of mice bearing tumors derived from ovarian cancer cells with elevated CCNE1 expression. Kaplan-Meier curve summarizing survival of mice injected with OVCAR5 (***A***) and ES2 cells (***B***). Female athymic nude mice were injected *i.p.* with OVCAR5 or ES2 cells (1×10^7 cells/mouse) for 5 days followed by administration of 30 mg/kg SNS-032 twice per week until animal died.
